# Highly Sensitive Graphene-Au Coated Plasmon Resonance PCF Sensor

**DOI:** 10.3390/s21030818

**Published:** 2021-01-26

**Authors:** Hongyan Yang, Mengyin Liu, Yupeng Chen, Ling Guo, Gongli Xiao, Houquan Liu, Jianqing Li, Libo Yuan

**Affiliations:** 1 School of Electronic Engineering and Automation, Guilin University of Electronic Technology, Guilin 541004, China; hyyang@guet.edu.cn (H.Y.); 19082203009@mails.guet.edu.cn (M.L.); 19082203004@mails.guet.edu.cn (Y.C.); liuhouq@guet.edu.cn (H.L.); lbyuan@guet.edu.cn (L.Y.); 2 Guangxi Key Laboratory of Automatic Detecting Technology and Instruments, Guilin University of Electronic Technology, Guilin 541004, China; 3 Guangxi Key Laboratory of Opto-Electronic Information Processing, Guilin 541004, China; 4 Guangxi Key Laboratory of Precision Navigation Technology and Application, Guilin University of Electronic Technology, Guilin 541004, China; xiaogl.hy@guet.edu.cn; 5 Guangdong-Hong Kong-Macao Joint Laboratory for Intelligent Micro-Nano Optoelectronic Technology, Foshan University, Foshan 528225, China; jqli@must.edu.mo

**Keywords:** photonic crystal fiber, surface plasmon resonance, optical fiber sensor, graphene

## Abstract

This paper presents a graphene-Au coated photonic crystal fiber (PCF) sensor in the visible regime. Designing a side-polish D-shaped plane over the PCF’s defect of the periodic air holes can effectively enhance the evanescent field. Graphene on gold can enhance the sensor’s sensitivity because it can stably adsorb biomolecules and increase the propagation constant of the surface plasmon polariton (SPP). Using the finite element method (FEM), we demonstrated that the sensing performance is greatly improved by optimizing the PCF’s geometric structural parameter. The proposed PCF sensor exhibited high performance with a maximum wavelength sensitivity of 4200 nm/RIU, maximum amplitude sensitivity of 450 RIU^−1^, and refractive index resolution of 2.3 × 10^−5^ RIU in the sensing range 1.32–1.41. This research provides a potential application for the design a new generation of highly sensitive biosensors.

## 1. Introduction

Surface plasmon resonance (SPR) fiber sensors have become a hot research topic in recent years due to their high sensitivity in detecting various biological and chemical components, and they have great applications in biomedical and life security fields. SPR is a phenomenon caused by free electron oscillation on metal-dielectric interfaces arising from incident electro-magnetic waves [1]. With the development of nanotechnology, SPR in biochemical sensing has drawn much interest from domestic and foreign scholars due to its real-time monitoring of unlabeled biological samples and high sensitivity [2,3,4,5].

Since the photonic crystal fiber (PCF) was first proposed by Yablonovitch et al. [6] and John et al. [7], several academic groups around the world have studied the theory and application of PCF. Compared to optical fiber, PCF has a distribution of air holes in the cladding region, extending infinitely along the fiber axis, and it has a flexible structure and many excellent properties, including endless single mode, high nonlinearity, high birefringence, large mode field size, an ease of filling material, low transmission loss, controllable dispersion, and so on [8,9,10]. It is proven that PCF is capable of replacing prisms, with a smaller footprint, simpler system integration, and higher cost-effectiveness [11,12]. 

With the PCF acting as a prism, the SPR can be excited and controlled, which is known as PCF-SPR sensing technology and is a high-performance sensor. In recent years, many PCF-SPR sensors based on microstructures have been reported. Plasmonic materials are, however, coated on the inner wall of air holes in these sensors and liquid samples are selectively impregnated into these air holes, which is very difficult. The D-type PCF-SPR sensors [13,14,15], which are polished along one side of the optical fiber and placed directly above the defective air holes, have been proposed to address these difficulties. The plasmonic material and sensing layer are placed on the top of the polished surface, and air holes of different sizes are placed at different positions of the PCF structure to control the propagation of light in a specific direction. In 2015, Rifat et al. [1] proposed a PCF biosensor coated with copper metal and externally with graphene. The structure has a maximum sensitivity of 2000 nm/RIU within the refractive index range of 1.33–1.37, and a sensing measurement accuracy of 5 × 10^−5^ RIU. In 2015, Luan et al. [16] proposed a SPR sensor with a maximum sensitivity of 3700 nm/RIU and a sensing measurement accuracy of 2.7 × 10^−5^ RIU within the refractive index range of 1.33–1.37. In 2017, Hasan et.al. [17], proposed an SPR-based PCF biosensor outside the PCF structure with a gold layer and the authors realized a maximum of 2200 nm/RIU for a sample range of 1.33–1.36. PCF-SPR technology plays an important role in real-time detection and analysis, environmental monitoring, chemical detection, food safety, gas detection, glucose monitoring, and other fields [18,19].

Two-dimensional materials composed of single-layer atoms have attracted a lot of interest since the first discovery of graphene in 2004 by Novoselov et al. [20] from the University of Manchester. Their unique electrical, optical, and mechanical properties make them promising materials for laser, photovoltaic, sensor, and medical applications [21,22,23,24,25]. For example, graphene’s high electrical conductivity, flexibility, strength, and lack of dangling bonds, make it easy to integrate into fiber optics. Secondly, graphene has no mass, a unique Dirac cone structure leading to zero bandgaps, and it has an ultra-fast migration speed (up to 200,000 cm^2^/V s) at room temperature and high specific surface area, all these contribute to the absorption of biomolecules, leading to highly sensitive biomolecule sensing. Therefore, in recent years, many scholars have studied graphene biosensors for their apparent advantages, high sensitivity, and label-free, real-time detection of biological molecules (protein, DNA, etc.) [26,27,28].

In this paper, an SPR sensor with graphene-Au coated on D-type PCF is proposed. The proposed PCF sensor consists of two layers of holes, and the second layer is missing two air holes, forming an asymmetric structure in the orthogonal direction of the fiber core and generating a birefringence effect. This design can not only obtain high sensitivity, but also realizes low loss transmission. This makes the method of detection easier and simpler since the sample can be detected by simply flowing or dripping onto the graphene-coated gold layer’s outer surface. Manufacturing, which is a better candidate for refractive index sensor applications, is much simpler.

## 2. Theoretical Analysis and Sensor Design

### 2.1. Sensing Principle

**Figure 1:**
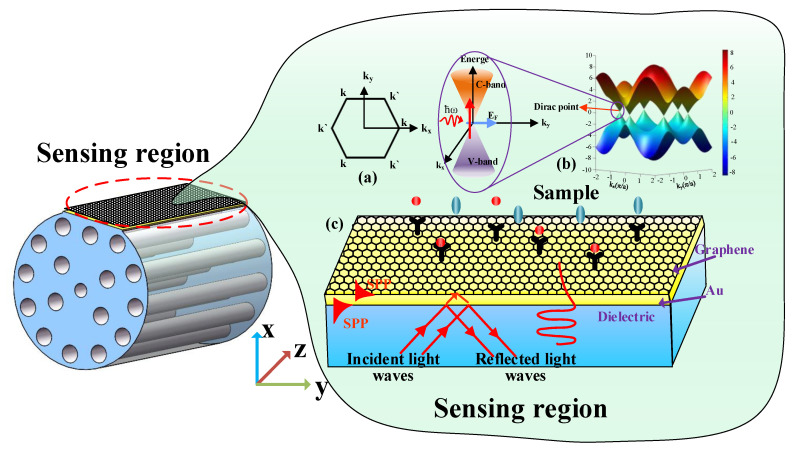
Surface plasmon resonance (SPR) sensing mechanism of D-type photonic crystal fiber (PCF) sensor. (**a**) The Brillouin zone of the graphene lattice. (**b**) Linear dispersion diagram of the band structure of single-layer graphene. (**c**) Schematic diagram of Graphene-Au sensing mechanism.

The principle of SPR is shown in Figure 1c. At the interface between the metal and medium, the evanescent wave generated by total reflection enters the metal layer and interacts with the free electrons to excite the surface plasmon wave (SPW), propagating on the surface of the metal layer. SPR is a kind of optical physical phenomenon. When the incident wavelength meets a certain value (resonance), most of the incident light is converted into the energy of SPW, resulting in the reflected light energy decreasing dramatically and the presence of a resonance peak in the reflection spectrum. At this time, the incident wavelength is called the resonance wavelength of SPR. By measuring the shift of the resonance wavelength, the sample’s refractive index (RI) on the surface of the metal layer can be obtained.

### 2.2. Kubo Model of Graphene

To optimize the sensitivity of the graphene-Au SPR sensor based on D-type PCF, it is necessary to know the optical property of graphene. As can be seen in Figure 1a, graphene is a single layer of carbon atoms in sp^2^ hybrid orbitals tightly packed into a two-dimensional honeycomb lattice structure material. k_x_ and k_y_ are the components of the wave vector k. Because k and k’ are symmetric and the conduction band and valence band of the two points in the Brillouin region are degenerate, the linear dispersion relation of the energy band of graphene is the result. Therefore, electrons can be regarded as massless relativistic particles, i.e., Dirac fermions, and the dispersion relation of two-dimensional electron energy is isotropic, which is called a Dirac cone [29]. Figure 1b shows the band structure of graphene, and the Dirac point region is enlarged in the inset. It is can be seen that the graphene is a kind of semimetallic material with zero band gap. The conduction band (C-band) and the valence band (V-band) are symmetrically conical and intersect at a point. This energy band structure satisfies the Dirac equation, instead of the Schrodinger equation satisfied by traditional metals or semiconductors, whose intersection point is called the Dirac point. The unique zero band gap, excellent mechanical properties, high thermal conductivity, great specific surface area, super broadband optical response spectrum, and strong nonlinear optical properties have significant advantages in the new type of optical and photoelectric sensing technology. Graphene can be transformed from semimetallic to metallic behavior by chemical doping or electrical gating, which largely depends on *μ* (the chemical potential) [24,30,31,32]. The random phase approximation of the dynamic optical response of graphene can be obtained from the complex Kubo equation, including inter-band and intra-band contributions [33,34]

(1) $$\sigma =\sigma_{\mathrm{int}ra}+{\sigma^{\prime }}_{\mathrm{int}er}+i{\sigma^{\prime \prime }}_{\mathrm{int}er}$$

(2) $$\sigma_{\mathrm{int}ra}=\sigma_{0}\frac{4\mu }{\pi }\frac{1}{\hbar \tau_{1}-i\hbar \omega }$$

where $\sigma_{0}=\frac{\pi e^{2}}{\left(2h\right)}$**,** $\tau_{1}$ is the intra-band transitions relaxation rate, ℏ is the constant of the reduced Planck’s, μ>0 is the chemical potential, and ω is the radian frequency. The inter-band contribution is

(3) $${\sigma^{\prime }}_{\mathrm{int}er}=\sigma_{0}\left(1+\frac{1}{\pi }\arctan\frac{\hbar \omega -2\mu }{\hbar \tau_{2}}-\frac{1}{\pi }\arctan\frac{\hbar \omega +2\mu }{\hbar \tau^{2}}\right)$$

and

(4) $${\sigma^{\prime \prime }}_{\mathrm{int}er}=-\sigma_{0}\frac{1}{2\pi }\ln\frac{{\left(2\mu +\hbar \omega \right)}^{2}+\hbar^{2}\tau_{2}^{2}}{{\left(2\mu -\hbar \omega \right)}^{2}+\hbar^{2}\tau_{2}^{2}}$$

where $\tau_{2}$ is the inter-band transitions rate of relaxation.

According to the above equation, the intra-band and inter-band photoconductivities of graphene are related to *μ* and *ω*. The *μ* of the doped graphene is controlled by the concentration of carrier n_0_ = ${\left(\mu /\hbar \upsilon \right)}^{2}/\pi$, which can be controlled by chemical doping or applied voltage. The *μ* = 0 of the pristine graphene has no intra-band contribution. Due to the appearance of the zero band gap, it can be seen from the theoretical expression and the experimental results of optical absorption that the intra-band optical conductivity $\sigma_{\mathrm{int}ra}$ in terahertz and far-infrared bands is dominant, while the total conductivity in near-infrared and visible bands mainly depends on the inter-band transition process [35]. Importantly, the intra-band contribution is related to the propagation of surface plasmon in graphene.

In the simulation, the relation between the graphene dielectric constant in the direction of the plane and the complex surface conductivity is as follows [36]:

(5) $$\varepsilon =1+\frac{i\sigma }{\varepsilon_{0}\Delta \omega }$$

The refractive index of graphene is as follows [37]:

(6) $$n_{g}=\sqrt{1+i\frac{\sigma }{\varepsilon_{0}\Delta \omega }}$$

where $\varepsilon_{0}$ is the vacuum permittivity and Δ = 0.34 nm is the thickness of graphene.

For multilayer graphene, thickness is calculated by t = 0.34 nm × L_G_ (L_G_ = 1, 2, 3…), where L_G_ is the number of layers. It has been proven that in the framework of the Fresnel coefficient calculation, as long as the consistency of graphene and experimental spectrum are added together, the distribution of graphene in the visible range can be simply estimated. The complex refractive index equation of graphene can be calculated [38,39]:

(7) $$n_{g}=3+\frac{iC_{1}\lambda }{3}$$

where *λ* (μm) is the wavelength and $C_{1}\approx 5.446\;\mu m^{-1}$ is implied by the opacity measurement by Nair et al. [38]. 

### 2.3. Drude–Lorentz Model

The Drude–Lorentz model leads to very small errors in a wide spectral range (λ≥630 nm), which is better than that of the Drude model [40]. We used the graphene-gold material coating on the PCF sensor in this paper, so the Drude–Lorenz model is more accurate. Here, we choose the gold layer thickness tg = 40 nm and the gold dispersion model Drude–Lorenz is:

(8) $$\varepsilon_{DL\left(Au\right)}\left(\omega \right)=\varepsilon_{\propto }-\frac{\omega^{2}{}_{D}}{\omega \left(\omega +j\gamma_{D}\right)}-\frac{\Delta \varepsilon \cdot \Omega_{L}^{2}}{\left(\omega^{2}-\Omega_{L}^{2}\right)+j\Gamma_{L}\omega }$$

where $\Omega_{L}$ is the oscillator strength, $\Gamma_{L}$ is the spectral width of the Lorentz oscillators, and Δε can be interpreted as a weighting factor [40]. Table 1 lists the detailed dielectric constants.

### 2.4. Sellmeier Equation

**Figure 2:**
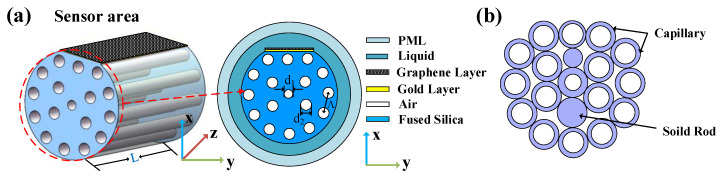
(**a**) Two-dimensional cross-section of the three-dimensional structure of the designed D-type PCF sensor (*Λ* = 1.8 μm, d = 0.4 *Λ*, t_g_ = 40 nm, and L_G_ = 3). (**b**) Stacked design of the PCF.

As shown in Figure 2b, the designed PCF could be manufactured by adding capillary and solid rods using a standard stack-and-draw system [41]. The background material of the sensor is fused silica, for which the Sellmeier equation below can obtain dispersion characteristics [42]:

(9) $$n\left(\lambda \right)=\sqrt{1+\frac{M_{1}\lambda^{2}}{\lambda^{2}-N_{1}}+\frac{M_{2}\lambda^{2}}{\lambda^{2}-N_{2}}+\frac{M_{3}\lambda^{2}}{\lambda^{2}-N_{3}}}$$

where *M*_1_ = 0.69616300, *M*_2_ = 0.407942600, *M*_3_ = 0.897479400, *N*_1_ = 0.00467914826, *N*_2_ = 0.0135120631, and *N*_3_ = 97.9340025.

### 2.5. Structural Design and Analysis

The PCF sensor is designed as seen in Figure 2. The cross-section of the D-type PCF sensor is shown in Figure 2a. The outer layer is the perfect matching layer (PML), and the second layer is the liquid layer (analyte). The material is fused silica, the yellow is the gold layer, and the black pattern is the graphene layer. The PCF structure consists of two air-hole rings. The first ring has two missing air holes and forms an asymmetric structure in the orthogonal direction of the fiber core and produces a birefringence effect. The air holes of the second ring are arranged in D shape with rotation angle of 30° between adjacent air holes. Designing a side-polish D-shaped plane over the PCF’s defect of the periodic air holes can effectively enhance the evanescent field. The smaller central air hole is designed in the sensor to make the evanescent wave penetrate the medium area and reach the metal–medium interface to excite more free electrons. On the other hand, it facilitates the phase matching coupling between the core-guide mode and the surface plasmon polariton (SPP) mode. In Figure 2a, the length of the sensing area is L = 1 mm, two adjacent air holes with diameter d = 0.4 *Λ* are placed with pitch size *Λ* = 1.8 μm, and the diameter of the scaled down air hole in the center is d_1_ = 0.2 *Λ*.

The sensor is surrounded by the sample to be tested, and the waveguide property and sensing performance are studied by COMSOL, the commercial software based on finite element. In this study, the circular anisotropic PML boundary condition was imposed, and by optimizing the mesh size and density, the convergence test was carried out, and the calculated results were accurate.

## 3. Results and Discussion

### 3.1. Dispersion Relation and Mode Field Distribution

**Figure 3:**
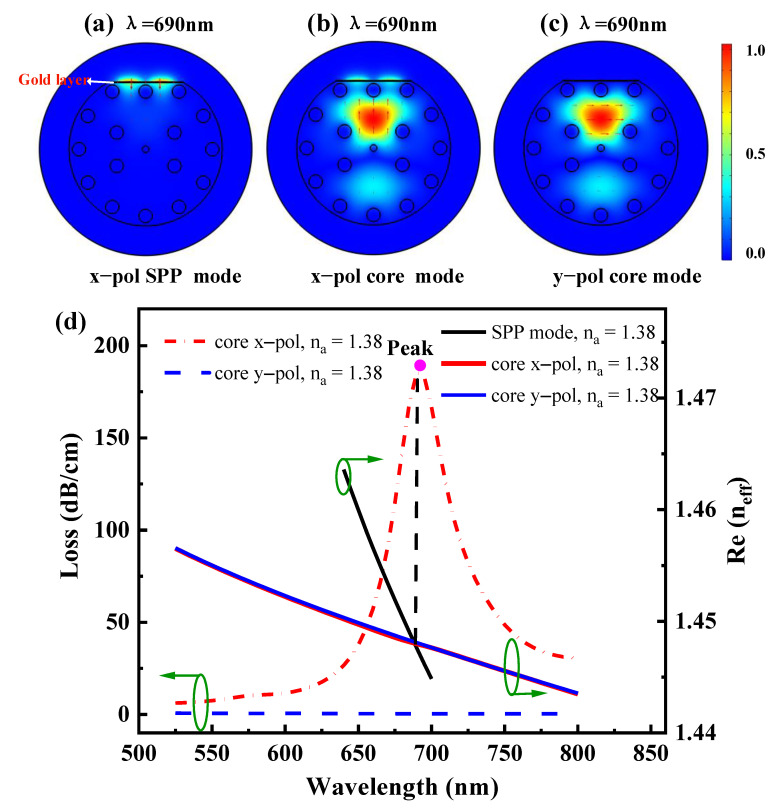
Mode field distribution of fundamental (**a**) x-pol surface plasmon polariton (SPP) mode, (**b**) x-pol core mode, and (**c**) y-pol core mode at 685 nm. (**d**) Relation of dispersion between the fundamental core-guided mode and SPP mode with *n_a_* = 1.38, *t_g_* = 40 nm, and L_G_ = 0.

Figure 3a–c describes the mode field distribution, which mainly helps us intuitively observe the coupling strength. The color bar represents the intensity distribution of the normalized mode field, and its value ranges from 0 (weak) to 1 (strong) and its color variance from blue to red. Figure 3d shows the dispersion curves of the SPP mode and core mode when RI is 1.38, t_g_ = 40 nm and L_G_ = 0. The X-axis is the wavelength, the right Y-axis is the real part of the effective refractive index, representing the light dispersion ability of the sensor. The left Y-axis is the attenuation constant per centimeter, which has the same change trend as the imaginary part curve of the effective refractive index and does not affect the wavelength sensitivity (S*_λ_*) evaluation. It represents the light absorption (loss) ability of the sensor.

Figure 3d shows that the solid and dashed lines in different colors represent different meanings. The solid black line represents the real part of the effective refractive index Re(n_eff_) of (a) x-pol SPP mode, the solid red line and the dotted red line represent the Re(n_eff_) and the confinement loss of 3b x-pol core mode, respectively, and the solid blue line and the dotted blue line are Re(n_eff_) and the confinement loss of Figure 3c y-pol core mode, respectively, which are at the wavelength of 690 nm. Figure 3a shows that in the x-pol SPP mode, there is almost no energy in the core and cladding, and all the energy is mainly concentrated near the gold layer, which cannot exist for a long time. Figure 3b shows the x-pol core mode. It can be seen that a large amount of energy from the core excitation to the cladding is coupled into the vicinity of the gold layer. At this time, SPR is the strongest. Figure 3c is the y-pol core mode. It can be seen that a large amount of energy is excited by the core to the cladding, but not coupled into the gold layer. That is because the y-pol core mode cannot produce the SPP mode, the mode field is only distributed in the PCF fiber. The reason is that when the electromagnetic wave propagates as a longitudinal wave in the metal at group velocity, it satisfies the phase matching, that is, the plasma frequency is equal to the evanescent wave frequency, and the corresponding excitation is the collective longitudinal vibration of the electron. At this time, the incident light is converted into SPW energy, resulting in a sharp decrease of reflected light energy and a confinement loss peak. Therefore, the x-pol SPP mode can excite SPR, indicating that the red dotted line corresponding to the intersection of the red solid line and the black solid line in Figure 3d has a peak value, while the y-pol SPP mode hardly excites SPR, indicating that the blue dotted line corresponding to the intersection of the blue solid line and the black solid line has no peak value. Therefore, the x-pol core mode is considered in the following discussion. COMSOL software can solve the effective refractive index of mode fields in a complex domain. The effective refractive index(n_eff_) of the mode can be expressed as n_eff_ = Re(n_eff_) + iIm(n_eff_). The confinement loss can be obtained by Equation (8) [43]:

(10) αloss=40πλIn10Im(neff)(dB/m)

where Im(neff) is the imaginary part of the effective refractive index.

### 3.2. Discussion on the Varying of Λ, and d_1_

**Figure 4:**
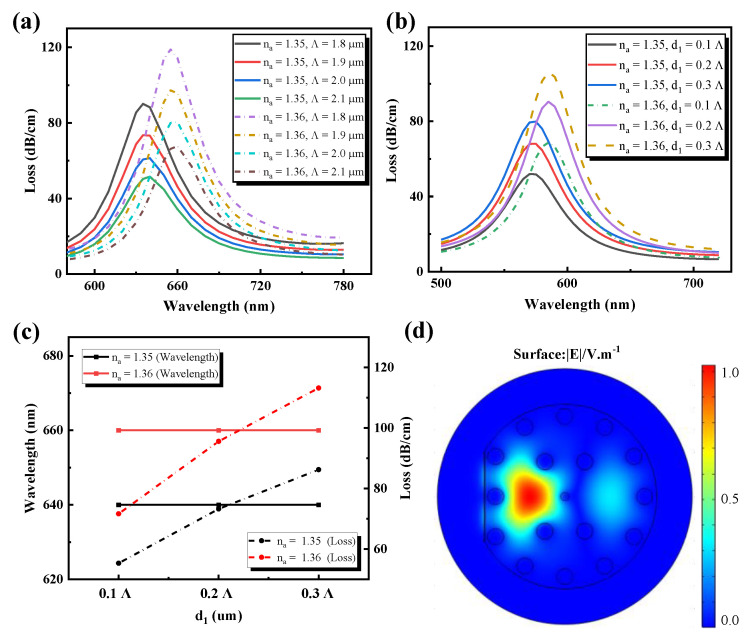
Influence of PCF structure parameters on the loss spectrum curve. (**a**) Air holes pitch, *Λ*; (**b**) Air holes diameter, d_1_; (**c**) Resonance wavelength varies with the diameter, d_1_; (**d**) d_1_ = 0.2 *Λ* optical field distribution.

The PCF-SPR sensor’s sensing capacity also depends on PCF structural parameters, such as pitch, *Λ,* and holes diameter, d_1_, which are shown in Figure 4. The impact of pitch *Λ* on the spectrum of loss are displayed in Figure 4a. As the sample RI varies from 1.35 to 1.36, the loss peak moves to the longer wavelength and simultaneously raises the loss depth. When the sample RI is unchanged, the loss of the resonant peak position remains unchanged, with n_a_ = 1.35 at 635 nm and n_a_ = 1.36 at 655 nm. It is obvious that the loss depth is decreasing, meaning that in the fiber core, light is more constrained. The maximum loss peaks for naval values of 1.35 and 1.36 were observed at 635 nm and 655 nm at 90 and 118.9 dB/cm, respectively, with parameters of *Λ* = 1.8 μm and L_G_ = 0. When *Λ* = 1.8 μm, the loss peak reaches the maximum and the SPR phenomenon is the most obvious. Therefore, the value of holes pitch *Λ* = 1.8 µm was selected as the optimal parameter of this design. Figure 4b,c analyzes the impact of the diameter of the smaller central air hole. For the increment in diameter d_1_, a related phenomenon is observed to appear as the *Λ* variance. It is clear that at d_1_ = 0.3 *Λ*, the maximum loss peaks are reached. The diameter d_1_ could not, however, be too high, leading to a reduction of the effective RI of the center of the fiber. The light would therefore leak from the core to the cladding area, which affects the efficiency of sensing. Therefore, d_1_ = 0.2 *Λ* was shown to be the optimized parameter and Figure 4d shows the optical field distribution for d_1_ = 0.2 *Λ.*

### 3.3. Discussion on the Varying Thicknesses of the Gold Layer

**Figure 5:**
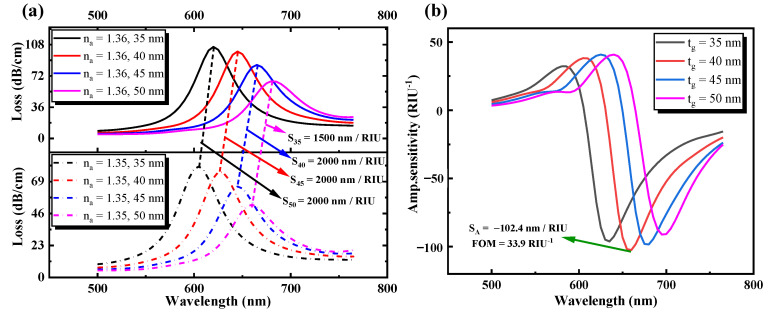
(**a**) Loss spectrum curve. (**b**) Amplitude sensitivity with different gold layer thicknesses from 35 to 50 nm.

Figure 5 illustrates the effect of the confinement loss and the sensitivity of amplitude on the efficiency of sensing with a difference in the thickness of the gold layer without graphene. In Figure 5a, the loss peaks decreased from 79.9 to 52.3 dB/cm for *n_a_* of 1.35 and from 105.48 to 65.9 dB/cm for *n_a_* of 1.36 with the increase in gold layer thickness from 35 to 50 nm. It can be seen that with a change in gold layer thickness from 35 to 50 nm, the loss peaks progressively downward; the thicker the gold layer, the greater is the loss of damping. Increasing the gold layer thickness would cause the peak loss to change towards a longer wavelength (red shift). The definition of the red or blue shift (movement to a shorter wavelength) can be determined by the equation [44] d_p_ = 1/kβ = *λ*/2πβ, where d_p_ is the evanescent wave’s penetration depth, and β and k are the decay constant and wave number, respectively. The equation n_eff_ = β/k_0_, k_0_ = 2π/*λ*, where k_0_ = 2π/*λ* is the wave number in the free space. This is because the incoming photon frequency is proportional to the depth of penetration [45]. Therefore, the longer the wavelength, the greater is the d_p_, the lower is the d_p_, and the shorter is the wavelength. Table 2 shows the corresponding sensitivity of the wavelength and the quality factor with the variation of gold layer thickness. 

The equation for calculating wavelength sensitivity, S*_λ_,* and amplitude sensitivity, S_A_, is as follows [46,47]:

(11) $$S_{\lambda }\left(nm/RIU\right)=\frac{\Delta \lambda_{peak}}{\Delta n_{a}}$$

and

(12) $$S_{A}\left(RIU^{-1}\right)=-\frac{1}{\alpha \left(\lambda ,n_{a}\right)}\frac{\partial \alpha \left(\lambda ,n_{a}\right)}{\partial n_{a}}$$

where ∆*λ_peak_* is the difference between the corresponding peak wavelength shifts, ∆*n_a_* is the variation of the sample’s RI, *α*(*λ*,*n_a_*) is the total loss of propagation at n_a_’s RI, *∂n_a_* is the alteration of the medium’s RI to be calculated, and *∂α*(*λ*,*n_a_*) is the difference of loss between two loss spectra. When the thickness of the gold layer is 35, 40, 45, and 50 nm, Equation (11) calculates the sensitivity of the wavelength as 1500, 2000, 2000, and 2000 nm/RIU, respectively. The higher the sensitivity and measurement precision of the SPR sensor, the better will be the performance of the sensor. However, the measurement accuracy would be decreased too much by the full width at the half limit (FWMH) of the resonance loss peak. Therefore, a consistency factor (FOM) evaluation parameter is introduced [48], and FOM = S(*λ*)/FWMH. In accordance with the equation, FOM is 24.6, 33.9, 30.8, and 31.3 while the thickness of the gold layer is 35, 40, 45, and 50 nm, respectively. Figure 5b also shows that the amplitude sensitivity ranges from 35 to 50 nm with the gold layer thickness. It is clearly apparent that the designed sensor structure displays 96.2, 102.4, 98.3, and 91.1 RIU^−1^ amplitude sensitivities, respectively, determined by the Equation (12). The maximum amplitude sensitivity and FOM can be obtained when the gold layer thickness is set to t_g_ = 40 nm. Therefore, t_g_ = 40 nm was used in the whole process to ensure better sensing performance.

### 3.4. Discussion on the Varying Layers of Graphene

**Figure 6:**
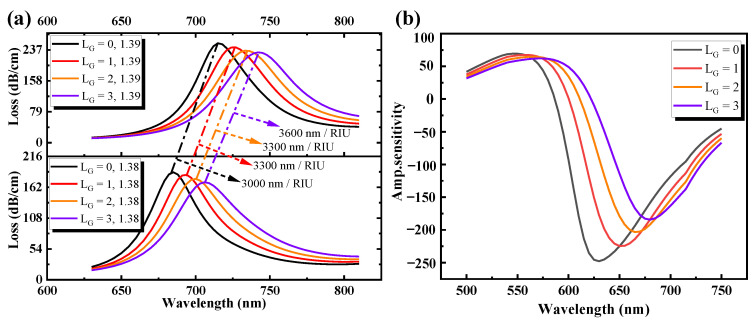
(**a**) Loss spectrum curve. (**b**) Amplitude sensitivity curve with different graphene layers from L_G_ = 0 to 3.

Figure 6 demonstrates the influence of loss value and amplitude sensitivity on the proposed PCF-SPR sensor with varying layers of graphene. From Figure 6a, it is evident that the resonance wavelength of the abscissa is redshifted with the increase of the sample RI and graphene thickness, and the value of the confinement loss peak in ordinate decreases gradually. At *n_a_* = 1.38, as graphene layers are expanded from zero layers (t = 0.34 nm × 0) to three layers (t = 0.34 nm × 3), the sensor loss value decreases gradually from 188.35 dB/cm to 170.78 dB/cm. Figure 6b also explains that with the graphene layers from 0 to 3, the amplitude sensitivity also decreases from 247.8 to 184.3 RIU^−1^. According to the sensitivity Equation (11), in the RI range of 1.38–1.39, as the number of graphene layers L_G_ increases from 0 to 3, the sensitivity is 3000 nm/RIU, 3300 nm/RIU, 3300 nm/RIU, and 3600 nm/RIU, respectively. The sensitivity of the gold layer coated with three layers of graphene is 20% higher than that without graphene. Although the sensitivity of the sensor increases with the increase of graphene layers, the value of the confinement loss peak and amplitude sensitivity decrease with the increase of graphene thickness due to the damping effect of graphene. Generally, because of the mechanical strength and chemical inertia of graphene, one layer or a double layer of graphene will avoid oxidation. The simulation in this paper was therefore carried out in terms of cost and sensing efficiency under the condition of three layers of graphene.

### 3.5. Discussion on the Varying of Sample RI (n_a_)

**Figure 7:**
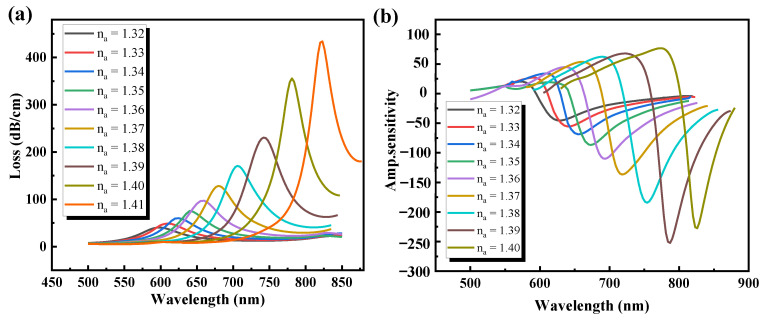
(**a**) Loss spectra curves at different *n_a_* with *Λ* = 1.8 μm, d = 0.4*Λ*, d1 = 0.2, t_g_ = 40 nm, and L_G_ = 3; (**b**) amplitude sensitivity.

The RI of the sensing medium has a great influence on the SPP mode, thus, the function of the PCF-SPR sensor would be influenced if the sample RI varied slightly. By a slight change in RI, a simple spectral shift is induced. As shown in Figure 7, the sensing characteristics accounting for the impact of the loss value and amplitude sensitivity on the RI sample were analyzed here. Figure 7a shows the loss spectrum curves for different RI values (1.32–1.41). From the figure, it is observed that when the RI increases from 1.32 to 1.41, with the increase of wavelength, the loss peak increases gradually, and the FWHM decreases gradually, and a red shift occurs. The principle of the red shift is comparable to that of study described in Section 3.3, since the difference of RI (∆RI) between the core and cladding decreases with the increase of sample RI, resulting in a greater amount of light penetrating through the cladding and interacting with the metal. The longer the wavelength, the higher the d_p_ and the greater the absence of confinement.

According to the wavelength corresponding to the loss peak, the confinement loss value of the sensor can be measured by Equation (10). For example, when *n_a_* = 1.32 and the wavelength is 0.60 μm, the peak value of resonance loss is 40.4 dB/cm; when *n_a_* = 1.33 and the wavelength is 0.61 μm, the calculated peak loss is 48.9 dB/cm. In the above analysis, the wavelength change is 0.01 μm, and the RI change is 0.01. According to Equation (11), the wavelength sensitivity is about 1000 nm/RIU. Similarly, when *n_a_* is changed from 1.32 to 1.41, the wavelength sensitivity is 4200 nm/RIU at 0.82 μm, and the peak value of resonant mode loss is 431 dB/cm when *n_a_* = 1.41. The results show that the maximum energy transfer from core mode to SPP mode is 0.82 μm when RI is 1.41.

The amplitude sensitivity obtained by varying the sample RI is shown in Figure 7b. The amplitude sensitivity increases with the increase in the sample RI, indicating that the interaction between the evanescent field and the SPP mode is enhanced, and the maximum sensitivity of the amplitude could reach 450 RIU^−1^. The average wavelength sensitivity is 1580 nm/RIU by wavelength interrogation in the RI range (*n_a_* = 1.32–1.37). Using the equation R(RIU) = ∆*n_a_* × (∆*λ_min_*)/(∆*λ_peak_*) = (∆*λ_min_*)/*S_λ_* [43], the sensor resolution is 6.3 × 10^−5^ RIU, where Δ*λ_min_* = 0.1 nm for the current spectrometer resolution technology. The average wavelength sensitivity is 3900 nm/RIU in the RI range (*n_a_* = 1.38–1.41), and the resolution of the sensor is 2.6 × 10^−5^ RIU. The maximum wavelength sensitivity is 4200 nm/RIU in the 1.32–1.41 RI range, and the highest sensor resolution is 2.3 × 10^−5^ RIU.

In the simulation process, we usually use the transition boundary condition of the surface charge ($\sigma_{s}$) to calculate graphene [28], and the thickness is 1 nm, that is, the surface charge density is the volume charge density. In order to facilitate a more accurate quantitative calculation, when we calculate the bulk charge density $\sigma_{v}=\frac{\sigma_{s}}{\Delta }=\frac{\sigma_{s}}{0.34nm}$ by using the empirical value of the thickness of single-layer graphene measured experimentally, i.e., 0.34 nm, it is found that when the grid density is divided into 25 layers and the minimum cell size of the grid is 0.0136 nm, the average wavelength sensitivity is 3900 nm/RIU, and the refractive index range is 1.38–1.41. 

Therefore, any slight change in the order of the RI of 10^−5^ can be observed. The maximum sensitivity and resolution of the wavelength in the proposed sensor was shown to be higher than those previously found in the literature (Table 3) [1,16,17,49].

**Figure 8:**
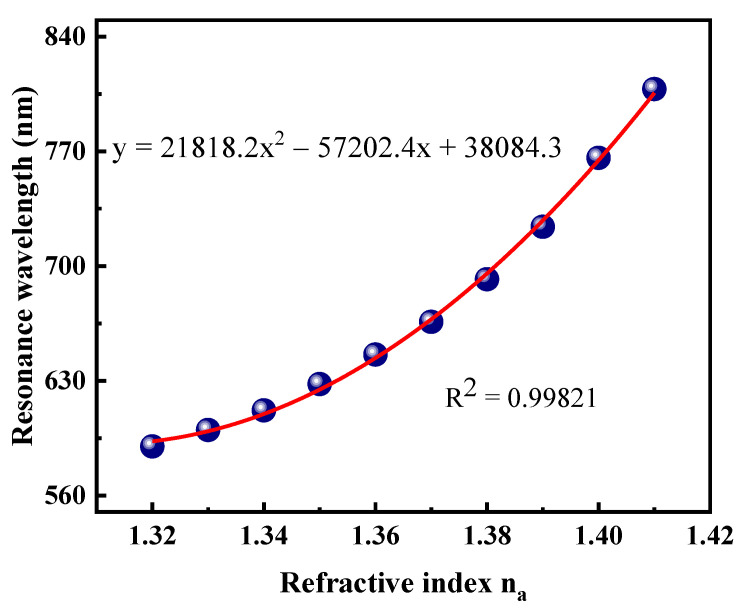
Polynomial fitting curve for sample refractive index (RI) variations from 1.32 to 1.41.

With the increase of RI, the change trend of the resonance wavelength is shown in Figure 8. For optimization of the sensor, the best polynomial fitting method is proposed. The polynomial fitting curve can be used to obtain the average sensitivity in the sensing range of 1.32–1.41. The red line in the figure represents polynomial fitting, and the blue balls are the resonance wavelengths. In the measurement range of 1.32–1.41, the correlation coefficient R^2^ = 0.99842 between sample RI and resonance wavelength. The corresponding polynomial regression equation is *λ* = 21174.2*n_a_*^2^ − 55377.8*n_a_* + 36807, where *n_a_* is the sample RI.

## 4. Conclusions

In conclusion, we proposed a graphene-Au coated PCF sensor in the visible regime based on SPR. The finite element method (FEM) was employed to investigate the fiber’s properties and sensing performance. By designing the structural geometric parameter of PCF and 2D material, high sensitivity was achieved in the proposed sensor. It was proven that graphene not only can be used to prevent Au oxidation, but can also enhance sensing performance. The proposed PCF-SPR sensor exhibited a maximum wavelength sensitivity of 4200 nm/RIU, a maximum amplitude sensitivity of 450 RIU^−1^, and a maximum resolution of 2.3 × 10^−5^ RIU in the sensing range of 1.32–1.41. The results provide a theoretical basis to design new high sensitivity biosensors that have great potential in detecting biomolecules, organic chemicals, and so on.

## Figures and Tables

**Table 1:** Parameter values of the Drude–Lorentz model.

	$\varepsilon_{\infty }$	$\omega_{D}/2\pi$	$\gamma_{D}/2\pi$	$\Omega_{L}/2\pi$	$\Gamma_{L}/2\pi$	Δε	Φ
		(*THz*)	(*THz*)	(*THz*)	*(THz*)		
Drude-Lorentz	5.9673	2113.6	15.92	650.07	104.86	1.09	14.521

**Table 2:** Quality factors corresponding to different gold layer thicknesses.

Gold Film Thickness (nm)	FWHM (nm)	S (nm/RIU)	FOM (RIU^−1^)
35	61	1500	24.6
40	59	2000	33.9
45	65	2000	30.8
50	64	2000	31.3

**Table 3:** Performance comparisons.

Analyle RI	Resonant Wavelength	Max Sensitivity	Resolution	Ref.
(RIU)	(nm)	(nm/RIU)	(RIU)	
1.33–1.37	520–770	2000	$5\times 10^{-5}$	Rifat et al. [1]
1.33–1.37	480–650	3700	$2.7\times 10^{-5}$	Luan et al. [16]
1.33–1.36	520–750	2200	$3.75\times 10^{-5}$	Hasan et al. [17]
1.33–1.35	480–750	2520	$3.97\times 10^{-5}$	Yang et al. [49],
1.32–1.41	500–800	4200	$2.3\times 10^{-5}$	This work

## Data Availability

Not applicable.

## References

[1] Rifat A.A., Mahdiraji G.A., Ahmed R., Chow D.M., Sua Y., Shee Y., Adikan F.M. (2015). Copper-Graphene-Based Photonic Crystal Fiber Plasmonic Biosensor. IEEE Photonics J..

[2] Knight J.C. (2003). Photonic Crystal Fibres. Nature.

[3] Hernández A.L., Casquel R., Holgado M., Cornago I., Fernández F., Ciaurriz P., Sanza F.J., Santamaría B., Maigler M.V., Laguna M.F. (2016). Resonant Nanopillars Arrays for Label-Free Biosensing. Opt. Lett..

[4] Jabbari S., Dabirmanesh B., Arab S.S., Amanlou M., Daneshjou S., Gholami S., Khajeh K. (2017). A Novel Enzyme Based SPR-Biosensor to Detect Bromocriptine as an Ergoline Derivative Drug. Sens. Actuators B Chem..

[5] Gollapalli R.P. (2020). Enhanced Sensitivity in Graphene-Based SPR Biosensors Using Electrical Bias. Opt. Lett..

[6] Yablonovitch E. (1987). Inhibited Spontaneous Emission in Solid-State Physics and Electronics. Phys. Rev. Lett..

[7] John S. (1987). Strong Localization of Photons in Certain Disordered Dielectric Superlattices. Phys. Rev. Lett..

[8] Yang J., Zhou L., Che X., Huang J., Li X., Chen W. (2016). Photonic Crystal Fiber Methane Sensor Based on Modal Interference with an Ultraviolet Curable Fluoro-Siloxane Nano-Film Incorporating Cryptophane A. Sens. Actuators B Chem..

[9] Cheng T., Duan Z., Liao M., Gao W., Deng D., Suzuki T., Ohishi Y. (2013). A Simple All-Solid Tellurite Microstructured Optical Fiber. Opt. Express.

[10] Wong W.C., Chan C.C., Chen L.H., Li T., Lee K.X., Leong K.C. (2012). Polyvinyl Alcohol Coated Photonic Crystal Optical Fiber Sensor for Humidity Measurement. Sens. Actuators B Chem..

[11] Verma R., Gupta B.D., Jha R. (2011). Sensitivity Enhancement of a Surface Plasmon Resonance Based Biomolecules Sensor Using Graphene and Silicon Layers. Sens. Actuators B Chem..

[12] Rifat A.A., Ahmed R., Yetisen A.K., Butt H., Sabouri A., Mahdiraji G.A., Yun S.H., Adikan F.R.M. (2017). Photonic Crystal Fiber Based Plasmonic Sensors. Sens. Actuators B Chem..

[13] Chen M., Lang T., Cao B., Yu Y., Shen C. (2020). D-Type Optical Fiber Immunoglobulin G Sensor Based on Surface Plasmon Resonance. Opt. Laser Technol..

[14] Weng S., Pei L., Wang J., Ning T., Li J. (2017). High Sensitivity D-Shaped Hole Fiber Temperature Sensor Based on Surface Plasmon Resonance with Liquid Filling. Photonics Res..

[15] Xie Q., Chen Y., Li X., Yin Z., Wang L., Geng Y., Hong X. (2017). Characteristics of D-Shaped Photonic Crystal Fiber Surface Plasmon Resonance Sensors with Different Side-Polished Lengths. Appl. Opt..

[16] Luan N., Wang R., Lv W., Yao J. (2015). Surface Plasmon Resonance Sensor Based on D-Shaped Microstructured Optical Fiber with Hollow core. Opt. Express.

[17] Hasan M.R., Akter S., Rifat A.A., Rana S., Ali S. (2017). A Highly Sensitive Gold-Coated Photonic Crystal Fiber Biosensor Based on Surface Plasmon Resonance. Photonics.

[18] Quan M., Tian J., Yao Y. (2015). Ultra-High Sensitivity Fabry-Perot Interferometer Gas Refractive Index Fiber Sensor Based on Photonic Crystal Fiber and Vernier Effect. Opt. Lett..

[19] Verma R., Gupta B.D. (2014). A Novel Approach for Simultaneous Sensing of Urea and Glucose by SPR Based Optical Fiber Multianalyte Sensor. Analyst.

[20] Novoselov K.S., Geim A.K., Morozov S.V., Jiang D., Zhang Y., Dubonos S.V., Grigorieva I.V., Firsov A.A. (2004). Electric Field Effect in Atomically Thin Carbon Films. Science.

[21] Pearce R., Iakimov T., Andersson M., Hultman L., Spetz A.L., Yakimova R. (2011). Epitaxially Grown Graphene Based Gas Sensors for Ultra Sensitive NO_2_ Detection. Sens. Actuators B Chem..

[22] He Q., Wu S., Yin Z., Zhang H. (2012). Graphene-Based Electronic Sensors. Chem. Sci..

[23] Yavari F., Koratkar N. (2012). Graphene-Based Chemical Sensors. J. Phys. Chem. Lett..

[24] Bao Q., Loh K.P. (2012). Graphene Photonics, Plasmonics, and Broadband Optoelectronic Devices. ACS Nano.

[25] Jiang Y., Miao L., Jiang G., Chen Y., Qi X., Jiang X.-f., Zhang H., Wen S. (2015). Broadband and Enhanced Nonlinear Optical Response of MoS_2_/Graphene Nanocomposites for Ultrafast Photonics Applications. Sci. Rep..

[26] Wu L., Chu H., Koh W., Li E. (2010). Highly Sensitive Graphene Biosensors Based on Surface Plasmon Resonance. Opt. Express.

[27] Xia F., Wang H., Xiao D., Dubey M., Ramasubramaniam A.J.N.P. (2014). Two-Dimensional Material Nanophotonics. Nat. Photonics.

[28] Llatser I., Kremers C., Cabellos-Aparicio A., Jornet J.M., Alarcón E., Chigrin D.N.J.P. (2012). Graphene-Based Nano-Patch Antenna for Terahertz Radiation. Photonics Nanostructures-Fundamentals. Appl..

[29] Jiang X.Q., Liu Z.B., Tian J.G. (2017). Progress in Optical Properties and Applications of Graphene. Prog. Phys..

[30] Wang F., Zhang Y., Tian C., Girit C., Zettl A., Crommie M., Shen Y.R. (2008). Gate-Variable Optical Transitions in Graphene. Science.

[31] Li Z., Henriksen E.A., Jiang Z., Hao Z., Martin M.C., Kim P., Stormer H., Basov D.N. (2008). Dirac Charge Dynamics in Graphene by Infrared Spectroscopy. Nat. Phys..

[32] Jiao S., Gu S., Yang H., Fang H., Xu S. (2018). Highly Sensitive Dual-Core Photonic Crystal Fiber Based on a Surface Plasmon Resonance Sensor with a Silver Nano-Continuous Grating. Appl. Opt..

[33] Falkovsky L., Pershoguba S. (2007). Optical Far-Infrared Properties of a Graphene Monolayer and Multilayer. Phys. Rev. B.

[34] Gusynin V., Sharapov S. (2006). Transport of Dirac Quasiparticles in Graphene: Hall and Optical Conductivities. Phys. Rev. B.

[35] Xiao G.L., Yang X.H., Yang H.Y., Dou W.Y., Xu J., Wei Q., Li H., Zhang F., Li Q., Chen Y. (2019). Plasma Refractive Index Sensor with Tunable Cross Tie-Shaped Graphene Array Structure. Acta Opt. Sin..

[36] He S., Zhang X., He Y. (2013). Graphene Nano-Ribbon Waveguides of Record-Small Mode Area and Ultra-High Effective Refractive Indices for Future VLSI. Opt. Express.

[37] Bo Y., Xin-Xin Y., Jing-Yue F., Yong-Dan H., Hua Q., Shi-Qiao Q. (2015). Tunable Terahertz Plasmon in Grating-Gate Coupled Graphene with a Resonant Cavity. Chinese Phys. B.

[38] Nair R.R., Blake P., Grigorenko A.N., Novoselov K.S., Booth T.J., Stauber T., Peres N.M., Geim A.K. (2008). Fine Structure Constant Defines Visual Transparency of Graphene. Science.

[39] Bruna M., Borini S. (2009). Optical Constants of Graphene Layers in the Visible Range. Appl. Phys. Lett..

[40] Vial A., Grimault A.-S., Macías D., Barchiesi D., De La Chapelle M.L. (2005). Improved Analytical Fit of Gold Dispersion: Application to the Modeling of Extinction Spectra with a Finite-Difference Time-Domain Method. Phys. Rev. B.

[41] Birks T., Atkin D., Wylangowski G., Russell P.S., Roberts P. (1996). 2D Photonic Band Gap Structures in Fibre Form. Photonic Band Gap Materials.

[42] Rifat A.A., Hasan M.R., Ahmed R., Butt H. (2017). Photonic Crystal Fiber-Based Plasmonic Biosensor with External SENSING approach. J. Nanophotonics.

[43] Hautakorpi M., Mattinen M., Ludvigsen H. (2008). Surface-Plasmon-Resonance Sensor Based on Three-Hole Microstructured Optical Fiber. Opt. Express.

[44] Mahfuz M.A., Hossain M., Haque E., Hai N.H., Namihira Y., Ahmed F. (2019). A Bimetallic-Coated, Low Propagation Loss, Photonic Crystal Fiber Based Plasmonic Refractive Index Sensor. Sensors.

[45] Al Mahfuz M., Hasan M.R., Momota M.R., Masud A., Akter S. (2019). Asymmetrical Photonic Crystal Fiber Based Plasmonic Sensor Using the Lower Birefringence Peak Method. OSA Contin..

[46] Islam M.S., Cordeiro C.M., Sultana J., Aoni R.A., Feng S., Ahmed R., Dorraki M., Dinovitser A., Ng B.W.-H., Abbott D. (2019). A Hi-Bi Ultra-Sensitive Surface Plasmon Resonance Fiber Sensor. IEEE Access.

[47] Akjouj A., Mir A. (2020). Design of Silver Nanoparticles with Graphene Coatings Layers Used for LSPR Biosensor Applications. Vacuum.

[48] Zhan Y., Li Y., Wu Z., Hu S., Li Z., Liu X., Yu J., Huang Y., Jing G., Lu H. (2018). Surface Plasmon Resonance-Based Microfiber Sensor with Enhanced Sensitivity by Gold Nanowires. Opt. Mater. Express.

[49] Yang X., Lu Y., Liu B., Yao J. (2017). Analysis of Graphene-Based Photonic Crystal Fiber Sensor Using Birefringence and Surface Plasmon Resonance. Plasmonics.

